# Diagnostic applicability of the circulating growth factors in preeclampsia

**DOI:** 10.1038/s41598-025-15314-z

**Published:** 2025-09-26

**Authors:** Ho Yeon Kim, Seoyoung Moon, Kwan Heup Song, Hai-Joong Kim

**Affiliations:** 1 Department of Obstetrics and Gynecology, Korea University College of Medicine, Seoul, Korea; 2 Department of Obstetrics and Gynecology, Ansan Hospital, College of Medicine, Korea University, 123 Jeukgeum-ro, Danwon-gu, 15355 Gyeonggido Republic of Korea

**Keywords:** Preeclampsia, Epidermal growth factor, Stem cell factor, Hepatocyte growth factor, Leukocyte inhibitory factor, Biomarkers, Diseases, Health care, Medical research

## Abstract

This study aimed to compare the levels of various growth factors in maternal serum and umbilical vein serum between individuals with preeclampsia (PE) and those with normotensive pregnancies. One hundred eight pregnant women were enrolled in this prospective study. Blood samples were taken at third trimester. 67 serum samples of preeclampsia and 41 normotensive mothers were collected. Umbilical vein serum samples of 27 preeclampsia and 38 normotensive pregnancies were collected. Serum levels of BDNF, EGF, FGF-2, HGF, LIF, NGF-beta, PDGF-BB, PlGF-1, SCF, and vascular endothelial growth factor (VEGF)-A and D were measured. EGF, HGF, LIF, and SCF levels were significantly higher in PE compared to normotensive group after adjustment for gestational age. There were no differences in growth factors in umbilical vein serum between two groups. EGF, NGF-beta and SCF were associated with the elevation of systolic and diastolic blood pressure. SCF showed a positive correlation with ALT and creatinine and a decreasing trend as gestational age advances. The ROC curve revealed cutoff value of 36.6 pg/mL in EGF, 23.5 in HGF, 2.78 in LIF and 2.95 in SCF (*p* < 0.001, *p* = 0.042, *p* < 0.001, *p* < 0.001). Circulating growth factors in PE may be responsible for development or consequence associated with the pathogenesis in PE especially elevated EGF, HGF, LIF and SCF.

## Introduction

Preeclampsia is a complex, multifactorial pregnancy disorder characterized by new-onset hypertension and proteinuria after 20 weeks of gestation. It is widely recognized as a placental-origin disease, arising from impaired trophoblast invasion that leads to inadequate remodeling of the spiral arteries. This results in reduced uteroplacental perfusion and subsequent fetal hypoxia and malnutrition. Recent evidence has emphasized not only the central role of the placenta but also the importance of maternal cardiovascular maladaptation in the development of this condition^1^.

A hallmark of preeclampsia is endothelial dysfunction, driven in part by an imbalance in angiogenic and anti-angiogenic factors. Among the most well-studied biomarkers are soluble fms-like tyrosine kinase-1 (sFlt-1), an anti-angiogenic factor, and placental growth factor (PlGF), a pro-angiogenic protein essential for placental vascular development. sFlt-1 antagonizes vascular endothelial growth factor (VEGF) and PlGF by binding to them and blocking their interaction with receptors. An elevated sFlt-1/PlGF ratio—indicative of excess anti-angiogenic activity—has emerged as a clinically valuable tool for the diagnosis and prognosis of preeclampsia, particularly in early-onset and severe cases^2,3^. Despite its high sensitivity and specificity, especially in differentiating preeclampsia from other hypertensive disorders of pregnancy, its predictive performance remains inconsistent, particularly in populations with comorbid conditions such as diabetes or autoimmune diseases^4,5^.

Beyond these established markers, attention has increasingly turned to other growth factors involved in vascular development and endothelial function, which may provide further insight into the pathogenesis of preeclampsia. VEGF and insulin-like growth factor-1 (IGF-1) are critical for angiogenesis during early placental development^6,7^. However, few studies have comprehensively evaluated these and other growth factors in both maternal and fetal circulations.

Emerging evidence points to additional candidates with potential relevance to preeclampsia. Brain-derived neurotrophic factor (BDNF), for example, plays a role in vascular remodeling and is implicated in implantation, placental formation, and fetal growth^8,9^. Factors such as maternal age, nutritional status, and exercise can influence BDNF levels. Leukemia inhibitory factor (LIF), a pleiotropic cytokine of the interleukin-6 family, is essential for implantation and pregnancy maintenance, and alterations in its circulating levels have been noted in pregnancy-induced hypertension^10,11^.

Epidermal growth factor (EGF) and hepatocyte growth factor (HGF) also contribute to trophoblast proliferation, survival, and invasion through their respective signaling pathways, including EGFR and c-Met, with HGF further mediating anti-inflammatory effects^12–14^. Stem cell factor (SCF), known for mobilizing endothelial progenitor cells, and nerve growth factor-beta (NGF-β), involved in angiogenesis and healthy pregnancy maintenance, have likewise been implicated in the regulation of vascular function during pregnancy^15–18^.

Given that preeclampsia stems from abnormal placental angiogenesis and vascular maladaptation, investigating these growth factors may offer deeper insights into its pathophysiology. In this study, we analyzed and compared a range of growth factors in maternal and fetal circulations between pregnancies complicated by preeclampsia and normotensive controls. By identifying distinct alterations in growth factor profiles, we aim to clarify their potential roles in the development of preeclampsia and contribute to the broader understanding of its underlying mechanisms.

## Methods

This prospective study was conducted at Korea University Ansan Hospital from September 2019 to September 2023. Patients were recruited from those who received prenatal care and delivered at Korea University Ansan Hospital, and included individuals between 23 and 41 weeks of gestation.

The study adhered to the principles of the Declaration of Helsinki. Written informed consent was obtained from all participants, and the study was approved by the Institutional Review Board of Korea University Ansan Hospital (IRB No. 2020AS0222). Patient demographics, laboratory results, and clinical information were collected from medical records.

Exclusion criteria included fetal malformations, chorioamnionitis, loss to follow-up, and underlying medical conditions such as chronic hypertension, chronic renal disease, and autoimmune disorders including antiphospholipid antibody syndrome and systemic lupus erythematosus.

Gestational age was determined by either first-trimester ultrasound (crown-rump length) or the first day of the last menstrual period. Small for gestational age was diagnosed postnatally as a birth weight below the 10th percentile for gestational age. Preeclampsia was diagnosed when systolic blood pressure was ≥ 140 mmHg or diastolic blood pressure was ≥ 90 mmHg after 20 weeks of gestation, accompanied by proteinuria. Severe features of preeclampsia were defined as follows^19^: systolic blood pressure ≥ 160 mmHg or diastolic blood pressure ≥ 110 mmHg on two occasions; thrombocytopenia (platelet count < 100,000/µL); liver enzyme levels twice the normal value; serum creatinine > 1.1 mg/dL or doubling of baseline; pulmonary edema; or cerebral/visual symptoms. In cases with severe features, proteinuria was not required for diagnosis. Preeclampsia is classified as early-onset when it occurs before 34 weeks of gestation, and as late-onset when it occurs at or after 34 weeks of gestation. After the diagnosis of preeclampsia, intravenous magnesium sulfate was administered before delivery and continued after delivery for 24 h.

### Sample collection

The growth factors analyzed in this study were collected and measured from maternal serum and umbilical vein serum, respectively. In normotensive pregnancies, peripheral blood was obtained during the third trimester as part of routine prenatal care. In cases of preeclampsia, samples were collected at the time of diagnosis, prior to the administration of magnesium sulfate for seizure prophylaxis or fetal neuroprotection. Aspartate aminotransferase (AST), alanine aminotransferase (ALT), creatinine, and uric acid levels were measured using an automated enzymatic assay on a clinical chemistry analyzer(Cobas 8000, Roche Diagnostics, Mannheim, Germany), according to the manufacturer’s protocol. Complete blood count was measured using an automated analyzer(XN series, Sysmex Corporation, Kobe, Japan). Serum concentrations of soluble fms-like tyrosine kinase-1 (sFlt-1) and placental growth factor (PlGF) were quantified using a validated electrochemiluminescence immunoassay (Elecsys^®^ platform, Roche Diagnostics, Mannheim, Germany) on the Cobas e analyzer series. The sFlt-1/PlGF ratio was calculated by dividing the measured concentration of sFlt-1 (pg/mL) by that of PlGF (pg/mL), as per manufacturer’s instructions. Umbilical vein serum was collected immediately after delivery. All samples were centrifuged at 3000 rpm for 10 min at 4 °C to obtain serum, which was then collected, aliquoted, and stored at − 80 °C until analysis.

### ProcartaPlex multiplex immunoassay method

A growth factor ProcartaPlex panel was performed according to manufacturer’s instructions (Invitrogen by ThermoFisher Scientific, US, catalog no; EXP110-12170-901). This panel includes BDNF, EGF, HGF, LIF, NGF-beta, PDGF-BB, PlGF-1, SCF, VEGF-A, and VEGF-D. The standard curves were prepared for assay (Bio-Plex Multiplex Immunoassay System, Bio-plex 200 System). The results were calculated with Bio-Plex Manager Software (Version 6.1 Build 727, Bio-Rad). Minimum detection range for each markers are as follows; 1.33, 2.51, 7.59, 3.52, 7.35, 4.88, 1.95, 1.10, 5.27, and 1.62 pg/mL.

### Statistical analysis

For normality distribution, Kolmogorov-Smirnov test was performed for all continuous variables including levels of growth factors. If a variable followed a normal distribution, it was analyzed using the Student’s t-test. In cases where the data were not normally distributed, the non-parametric Mann–Whitney U test was used instead. For categorical variables, comparisons between groups were performed using the chi-square (χ^2^) test. When expected cell frequencies were small (typically < 5), Fisher’s exact test was applied to ensure valid results. Pearson or Spearman rank correlation coefficient was used for the analysis of correlation, and the statistical significance of this study was based on *P* value < 0.05. A receiver-operating characteristic (ROC) curve was used to determine the relationship between the sensitivity and the specificity, and to select the best cut-off concentrations of serum growth factors for the prediction of PE. Statistics were performed using SPSS version 20.0 (SPSS Inc., Chicago, IL., USA) and Graphpad Prism version 10.0 (Graphpad Software LLC, Boston, MA., USA).

## Results

Among total of 108 women, 67 women had preeclampsia while 41 women were normotensive without proteinuria. Umbilical vein serum was obtained from 27 women with preeclampsia and 38 from normotensive pregnancies.

Table 1 demonstrates maternal characteristics and obstetric outcomes. Gestational age at birth was significantly lower in PE group. There were no significant differences in maternal age, nulliparity and rate of diabetes and use of steroid. BMI, Systolic blood pressure (SBP), diastolic blood pressure (DBP), serum creatinine and the rate of magnesium use were significantly high in preeclampsia group (Table 2). The use of tocolytics were more frequent and CRP levels were higher in normotensive group. The birthweight was significantly smaller in PE group therefore the rate of SGA was significantly high in this group.

**Table 1:** Maternal characteristics and obstetric outcomes.

	Preeclampsia (*n* = 67)	Normal (*n* = 41)	*P* value
Age (year)	34.4 ± 5.2	33.1 ± 4.6	0.196
Gestational days at birth	246(232–256)	266(239–270)	0.002
Nulliparity (%)	64.2	65.9	> 0.999
Abortion history(%)	32.8	34.1	> 0.999
HDP history(%)	10.8	2.4	0.148
ART(%)	18.4	14.6	0.500
Severe features(%)	75		
Thyroid disease(%)			0.395
Hypo	6.0	12.2	
Hyper	1.5	0	
Diabetes(%)			0.382
GDM	10.4	19.5	
DM	6.0	7.3	
Placenta previa(%)	5.8	7.3	> 0.999
Malpresentation(%)	21.2	14.6	0.590
NRFHR(%)	19.2	12.2	0.408
Vaginal delivery(%)	17.0	34.1	0.085
Multiple pregnancy(%)	10.4	17.1	0.381
PROM(%)	6.0	22	0.092
Preterm labor(%)	6.0	14.6	0.175
Tocolytics(%)	0	12.2	0.019
Previous csec (%)	17.3	29.3	0.214
Magnesium use(%)	40.4	9.8	< 0.001
Antibiotics (%)	17	31.7	0.135
Steroid use(%)	44.7	29.3	0.186
SGA(< 10%) (%)	55.6	14.6	< 0.001
SGA(< 5%) (%)	30.8	12.2	0.046
LGA(> 90%) (%)	3.8	7.3	0.651
Birthweight (g)	2119 ± 615	2699 ± 940	< 0.001
Gender male (%)	48.9	46.3	0.648
APGAR score at 5 min < 7	10.2	14.6	0.539

ART artificial reproductive technology, GDM gestational diabetes, DM overt diabetes, NRFHR non reassuring fetal heart rate, PROM premature rupture of membranes, csec cesarean section, SGA small for gestational age, LGA large for gestational age.

^a^*P*-values calculated by student t-test or Mann-Whitney Wilcoxon test.

**Table 2:** Clinical parameters in preeclampsia and normotensive pregnancy.

	Preeclampsia (*n* = 67)	Normal (*n* = 41)	*P* value
BMI(g/m^2^)	31(27–34)	28(25–30)	0.026
SBP (mmHg)	157(142–166)	122(112–129)	< 0.001
DBP (mmHg)	99(90–109)	76(66–82)	< 0.001
AST (U/L)	19(15–29)	19(14–23)	0.839
ALT (U/L)	14(9–21)	11(8–17)	0.481
Serum Creatinine (mg/dL)	0.6(0.52–0.70)	0.52(0.48–0.55)	< 0.001
Uric acid (mg/dL)	6.3 ± 1.4	4.8 ± 1.4	0.086
Platelet count (10^3^/µL)	208(167–256)	238(196–278)	0.133
C-reactive protein(mg/dL)	0.26(0.20–0.42)	0.33(0.18–0.67)	0.033
sFlt-1/PlGF ratio	62.6(30.4–180.4) (36)^*^	7.4(6.1–25.1) (4)^*^	0.114

BMI body mass index, SBP systolic blood pressure, DBP diastolic blood pressure, AST aspartate aminotransferase, ALT alanine aminotransferase, sFlt-1 soluble fms like tyrosine kinase, PlGF placental growth factor,

*P*-values calculated by student t-test or Mann-Whitney Wilcoxon test.

*number of patients measured.

**Fig. 1:**
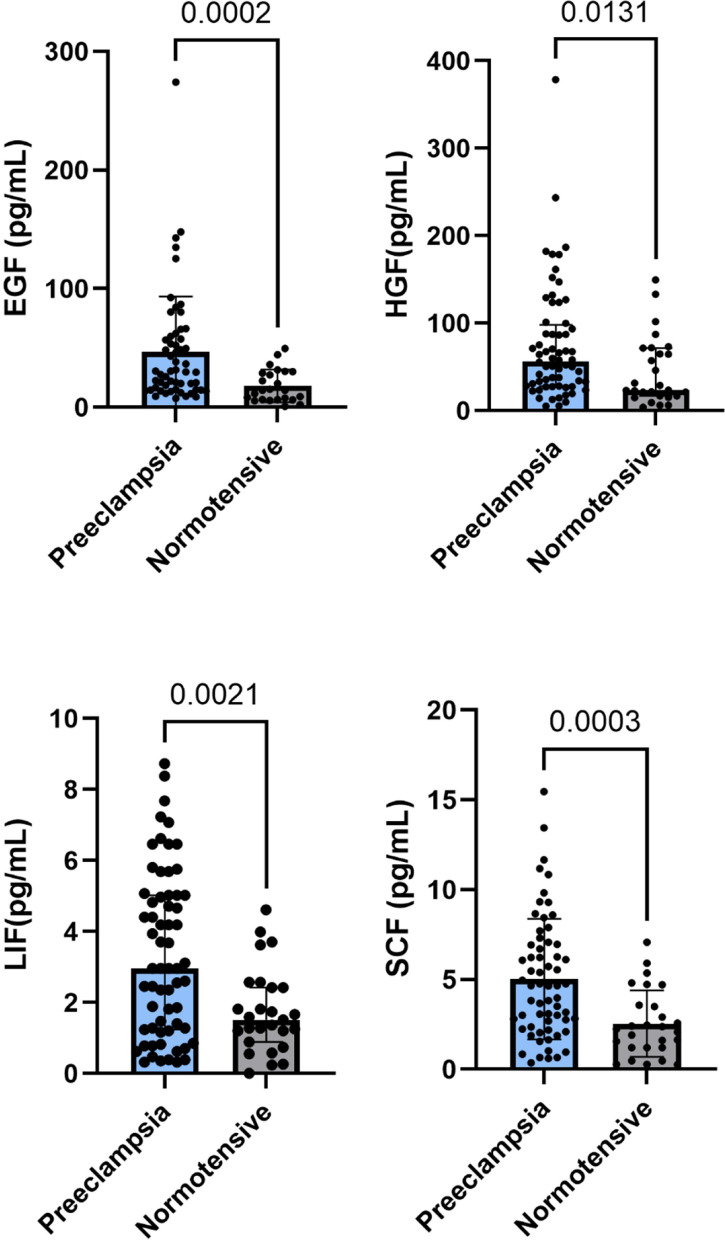
Circulating EGF, HGF, LIF, and SCF comparison between preeclampsia and normotensive mothers.

Among various growth hormones, maternal circulating EGF, HGF, LIF and SCF were significantly higher than those in normotensive group (Fig. 1). Even after adjusting for gestational weeks, these hormones still showed significant differences (Table 3). There were no significant differences in umbilical vein growth factors between two groups. Levels of EGF(*p* = 0.006), HGF(*p* < 0.001), NGF-β(*p* < 0.001), PDGF-BB(*p* = 0.028), SCF(*p* < 0.001), VEGF-A(*p* < 0.001) and D(*p* = 0.001) in umbilical vein serum were significantly higher compared to maternal serum.

**Table 3:** Various growth hormone concentrations in maternal and umbilical cord serum.

	Maternal blood serum (pg/mL)				Umbilical vein serum (pg/mL)		
	Preeclampsia (*n* = 67)	Normotensive (*n* = 41)	*p*-value^b^	*p*-value ^a, b^	Preeclampsia (*n* = 27)	Normotensive (*n* = 38)	*p*-value^b^
BDNF	5.5(3.2–11.2)	4.7(2.7-8.0)	0.195	0.730	3.5(2.6–6.5)	5.5(2.4–10.6)	0.075
EGF	30.5(15–60)	14.3(6.5–28.9)	< 0.001	0.041	60.9(39.7–142.8)	50.4(25.3–86.4)	0.445
FGF-2	2.3(1.3-4)	1.3(1−1.6)	0.240	Not available	2(1.2–3.8)	3.1(1.5–5.0)	0.858
HGF	56.6(28.1–98.3)	23.5(16.8–71.3)	0.013	0.036	132.9(53.9−322.1)	206.9(147.9–301.8)	0.203
LIF	3(1.2−5.0)	1.5(0.9–2.4)	0.002	0.009	3.1(2.6−5)	3.6(2.6–5.2)	0.680
NGF-beta	0.42(0.2–0.6)	0.31(0.2–0.45)	0.103	0.097	0.9(0.11−1.0)	0.9(0.6–1.3)	0.853
PDGF-BB	28.8(11.5–44.9)	9.6(7.4–14.5)	0.001	0.341	113.9 ± 259.4	192.3.6 ± 420.2	0.600
PlGF-1	40.5(30.7–51)	36.4(31.6–77.9)	0.890	0.343	29.6(19–51)	25.4(23.8–39.8)	> 0.999
SCF	4.6(2.3–6.9)	2.2(1.2–3.6)	< 0.001	< 0.001	19.4(10.7–28.0)	23.1(12.8–34.8)	0.578
VEGF-A	19.6 (12−34.7)	16.8(9.4–27)	0.256	0.421	455.3(166–1006)	693.3(481.6–1193.6)	0.445
VEGF-D	4.7(2.9–6.5)	2.0(1.2–4.4)	0.009	0.187	6(3.6–8.6)	5.9(3.2–11.9)	> 0.999

BDNF brain derived neutrotrophic factor, EGF epidermal growth factor, FGF-2 fibroblast growth factor-2, HGF hepatocyte growth factor, LIF leukemia inhibitory factor, NGF–beta nerve growth factor-beta, PDGF-BB platelet derived growth factor-BB, SCF stem cell factor, VEGF-A vascular endothelial growth factor-A, VEGF-D vascular endothelial growth factor-D.

^a^ Adjusted for gestational age.

^b^*P*-values calculated by student t-test or Mann-Whitney Wilcoxon test.

Table 4 demonstrates a comparison of maternal growth factors between preeclampsia with and without severe features. None of growth factors demonstrated differences between two groups. When comparing early and late preeclampsia, the growth factors showed no significant differences (Table 5).

**Table 4:** Maternal serum growth factor stratified by severe features.

	Severe features (*n* = 47)	No severe features (*n* = 15)	*P*-value
BDNF	4.6(2.9–8.2)	7(5–12)	0.236
EGF	29.8(14.9–54)	46(20.8–80)	0.524
HGF	26.6(30.8–101)	51(14.6–71)	0.942
LIF	3.1 ± 2.2	3.9 ± 2.8	0.245
NGF-beta	0(0−0.59)	0.3(0–1)	0.931
PDGF-BB	30(10–54)	29(19–41)	1.000
PlGF-1	44(31–51)	33(27–51)	0.847
SCF	5.2 ± 3.3	4.7 ± 3.5	0.674
VEGF-A	20(11.1–35.6)	20.3(12–68.2)	0.886
VEGF-D	5(3−6.3)	5(3–9)	0.978

All values are in pg/mL.

BDNF brain derived neutrotrophic factor, EGF epidermal growth factor, FGF-2 fibroblast growth factor-2, HGF hepatocyte growth factor, LIF leukemia inhibitory factor, NGF–beta nerve growth factor-beta, PDGF-BB platelet derived growth factor-BB, SCF stem cell factor, VEGF-A vascular endothelial growth factor-A, VEGF-D vascular endothelial growth factor-D.

^a^*P*-values calculated by Mann-Whitney Wilcoxon test.

**Table 5:** Maternal serum growth factors in early and late preeclampsia.

(pg/ml)	Early (*n* = 27)	Late (*n* = 63)	*P* value^a^
BDNF	4.0(2.5–9.1)	5.9(3.9–8.1)	0.130
EGF	20.1(12–38)	27.3(14.3–50)	0.631
HGF	56.3(29.6–109)	50(23–87)	0.766
LIF	2.6 ± 1.9	3 ± 2.3	0.411
NGF-beta	0.31(0-0.45)	0.18(0–0.56)	1.000
SCF	4.8 ± 3.5	4.1 ± 2.9	0.311
PDGF-BB	19(8.5–42)	25(8.7–37.0)	0.684
VEGF-A	19.2(12–36)	18.6(11.2–29.9)	0.874
VEGF-D	5(1.6-5)	3.8(2.0–6.0)	0.436

BDNF brain derived neutrotrophic factor, EGF epidermal growth factor, HGF hepatocyte growth factor, LIF leukemia inhibitory factor, NGF–beta nerve growth factor-beta, PDGF-BB platelet derived growth factor-BB, SCF stem cell factor, VEGF-A vascular endothelial growth factor-A, VEGF-D vascular endothelial growth factor-D.

^a^*P*-values calculated by Mann-Whitney Wilcoxon test.

**Fig. 2:**
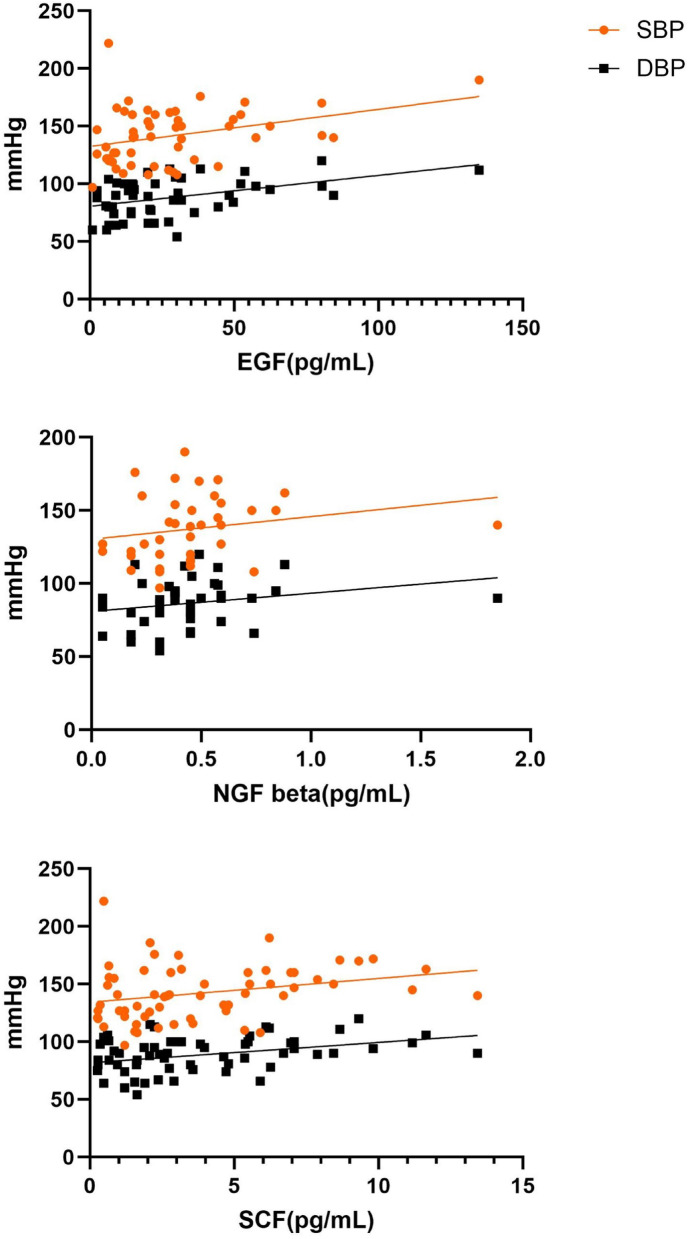
Correlation between EGF, NGF-beta and SCF with systolic and diastolic blood pressure.

**Fig. 3:**
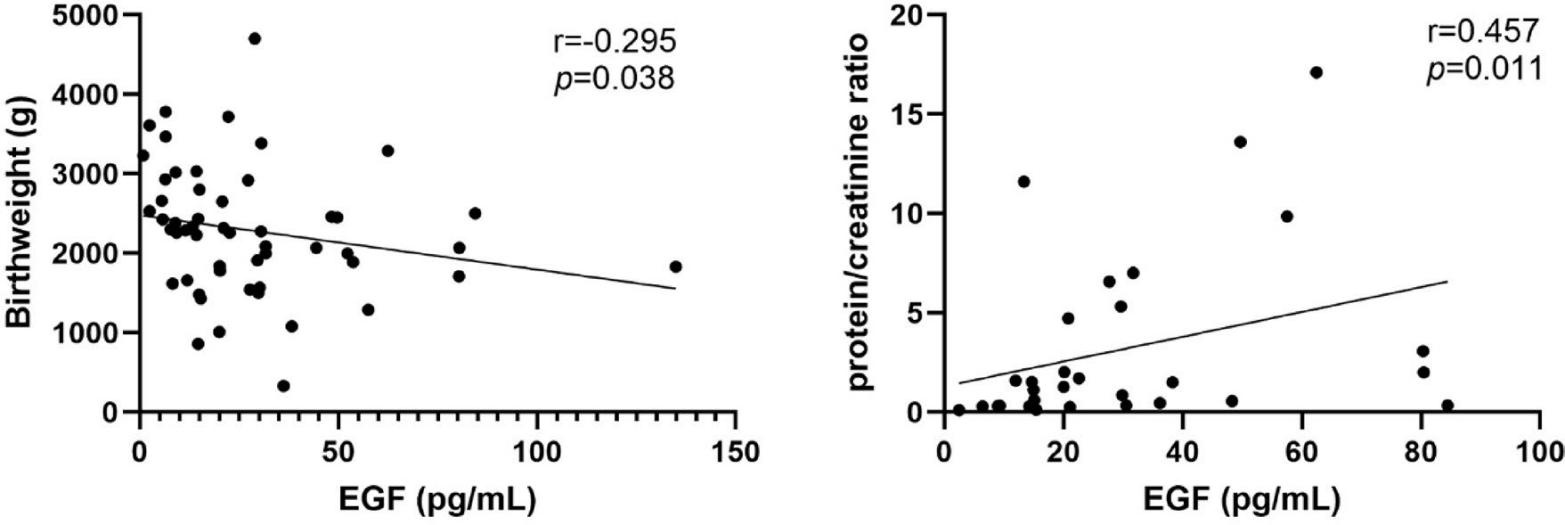
Correlation between EGF and birthweight and maternal urinary protein-creatinine ratio.

**Fig. 4:**
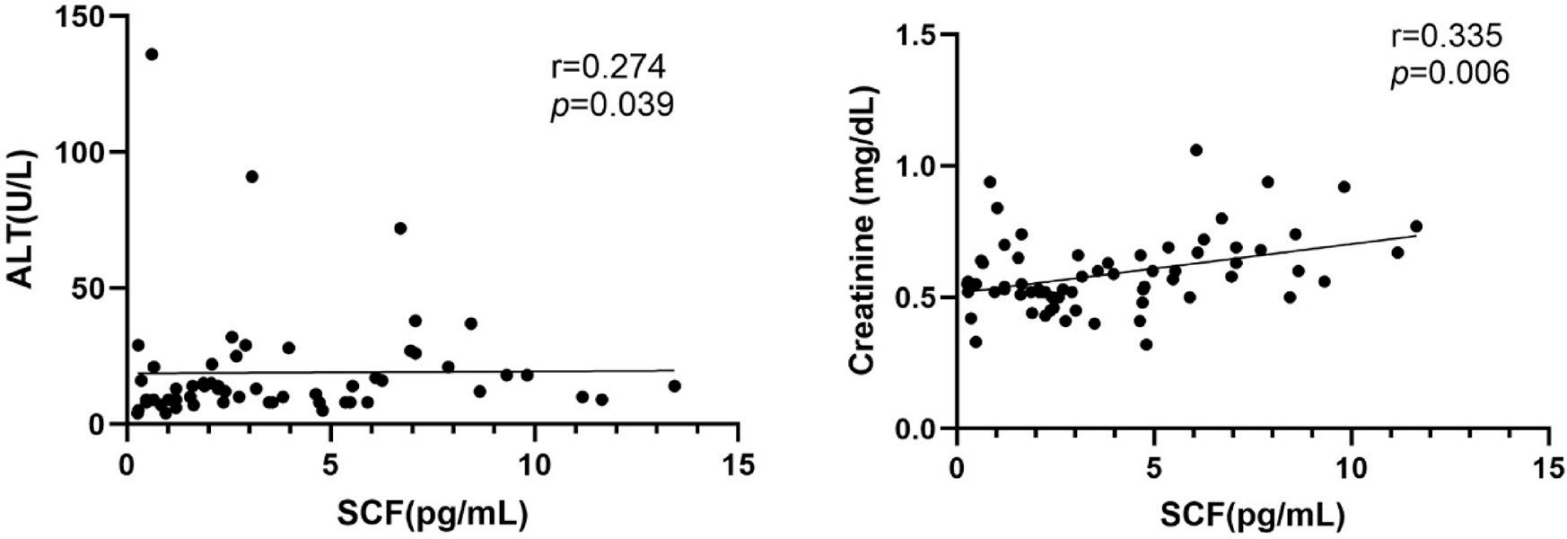
Correlation between SCF with maternal serum ALT and creatinine.

There was a positive association of serum EGF, NGF-beta and SCF levels with SBP and DBP (Fig. 2). SCF levels was inversely correlated with gestational days. EGF levels showed negative correlation with birthweight and positive correlation with protein creatinine ratio (Fig. 3). Circulating LIF levels showed positive association with platelet count. There were positive association of SCF and serum creatinine and ALT (Fig. 4).

Circulating BDNF was positively correlated with EGF, HGF, LIF, SCF, VEGF-D and PDGF-BB. EGF was positively correlated with BDNF, HGF, LIF, PDGF-BB, SCF. HGF was positively correlated with LIF, PDGF-BB, SCF and VEGF-D. LIF was positively correlated with PDGF-BB, SCF, and VEGFD. NGF-beta was positively correlated with VEGF-A. SCF was positively correlated with VEGF-A and VEGF-D.

**Fig. 5:**
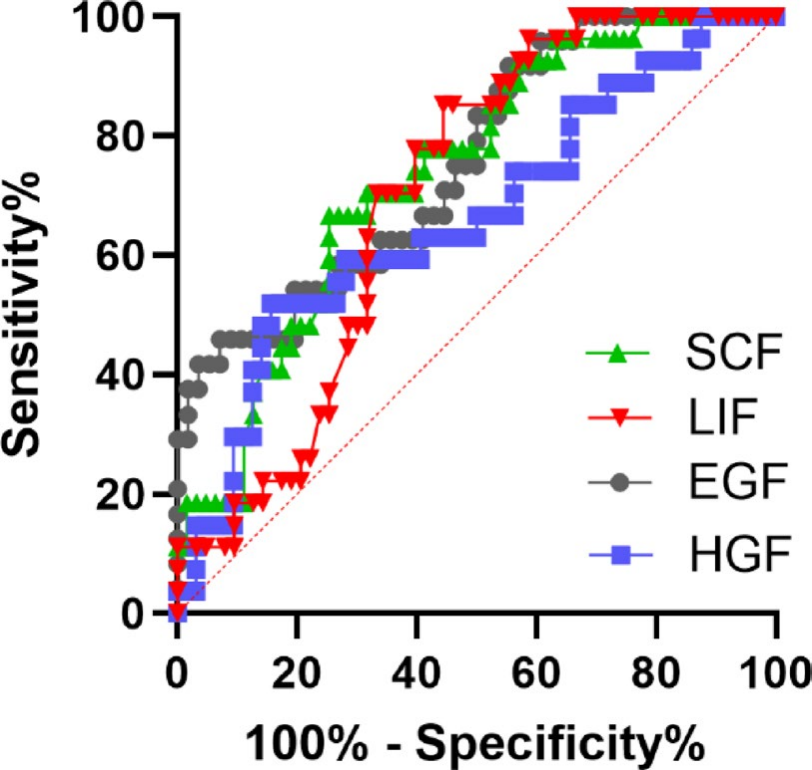
Receiver-operating characteristic curve showing the diagnostic value of EGF, HGF, LIF and SCF in preeclampsia.

The ROC curve analysis results are shown in graph (Fig. 5). The area under the ROC curve for EGF, HGF, LIF and SCF are 0.757 (95% CI 0.644–0.870), 0.738 (95% CI 0.633–0.844), 0.664 (95%CI 0.539–0.790) and 0.702 (95%CI 0.596–0.808) respectively. The ROC curve revealed sensitivity 47.3%, specificity 91% at cutoff value of 36.6pg/mL in EGF, 87.3% and 45% at cutoff value of 23.5 pg/mL in HGF and 63.6% and 77% at cutoff value of 2.78 pg/mL in in LIF and 78.2% and 32% at cutoff value of 2.95 pg/mL in SCF (*p* < 0.001, *p* = 0.042, *p* < 0.001, and *p* < 0.001).

## Discussion

Our study aimed to investigate changes in various growth hormones by measuring them in maternal blood and umbilical venous blood and comparing these hormones between preeclamptic and normotensive pregnant women. EGF, HGF and LIF and SCF levels were significantly elevated in preeclampsia. Various growth hormones in blood were significantly correlated with clinical factors related to preeclampsia such as blood pressure, maternal body mass index, urinary protein/creatinine ratio and maternal platelet. However various growth factors in umbilical cord blood which showed significantly higher levels than maternal blood, demonstrated no significant differences between preeclamptic and normotensive mothers.

Serum LIF in first trimester was significantly lower in women who were conceived after ART and later developed preeclampsia in previous research using bioinformatics and expressed lower in infertile endometrium^11,20^. Regardless of the inconsistent expression of LIF, it has been shown that LIF increases adhesion molecules via the JAK/STAT3 pathway, inducing inflammation and leading to endothelial dysfunction. LIF also plays a role in reducing nitric oxide synthesis^21^. This evidence supports the elevation of LIF observed in preeclampsia in our study.

While EGF and EGFR are known to be impaired in preeclampsia and its expression is reduced in preeclampsia^22,23^, another study displayed increased EGF in PE^24^, circulating levels of EGF in pregnancy showed inconsistent results. EGFR signaling increased PlGF secretion and positively regulates sFlt-1^22,25,26^. Heparin-binding EGF expression is decreased in PE placentas^22^. These alteration might be attributed to specific genetic polymorphism in the EGF gene in preeclampsia and low birthweight^27^. Regardless of the controversy surrounding changes in EGF levels in the blood, our results can be supported by previous findings that suggest villous trophoblast cells are likely the primary source of circulating EGFL7 in pregnant women. Elevated levels of EGFL7 have been observed in preeclampsia (PE) patients, supporting earlier reports of trophoblast damage due to hypoxia and the release of syncytiotrophoblast membranes into the maternal blood stream^28^. Furthermore, EGF–EGFR signaling has been shown to play a role in vascular regulation by mediating vasoconstriction and arterial hypertension^29^. Our results, which show a positive correlation between EGF and preeclampsia features (including the urinary protein-creatinine ratio and systolic and diastolic blood pressure), further support this evidence.

One study demonstrated that HGF is released when human vascular cells are stressed and is involved in vascular cell repair response^30^. HGF elevation in PE is likely to be explained by this function of HGF. Another study on urinary HGF revealed that this growth factor is increased in early gestation later developing preeclampsia^31^.

SCF was reported to regulate the proliferation of placental hematopoietic cells and be associated with preterm birth^8,15^. Previous studies have revealed increased circulating levels of SCF and other cytokines in postpartum hemorrhage, suggesting a peripheral proinflammatory phenomenon in PPH and lower levels of this hormone, implying endothelial dysfunction^7^. However, the role of this hormone in preeclampsia remains unknown; nevertheless, our study confirmed its increase in women with preeclampsia. One plausible theory for the elevated SCF might be induced by hypoxia, which is one of the pathophysiological phenomena of preeclampsia^9^.

There have been inconsistent results of circulating NGF^32^. Our study showed that NGF-beta levels in maternal blood and cord blood levels demonstrated no difference between preeclampsia and normotensive. However, subgroup analysis showed positive correlation with NGF levels and systolic and diastolic blood pressure in line with a previous research that placental expression also demonstrated positive correlation with systolic blood pressure^33^.

The concentrations of growth factors in umbilical vein were significantly higher than peripheral blood serum of mothers. Cord serum was known to contain rich sources of growth factors and cytokines with wound healing and anti-inflammatory properties^34^. Their utility is demonstrated by evidence in regenerative medicine^35^. The abnormal levels of growth factors such as PlGF was known to be associated with bronchopulmonary dysplasia in newborn^36^. However we observed no difference in growth hormone levels in cord blood between preeclampsia and normotensive pregnancy that neurodegenerative and vascular protection were kept through placenta for fetus development.

The variations in sensitivity and specificity of these markers may be due to factors such as the intrinsic properties of the markers themselves, their biological variability in preeclampsia. Sensitivity may be low if the markers are not consistently elevated across all individuals with preeclampsia. On the other hand, high specificity suggests that the markers are good at identifying individuals without the disease. Additionally, variations in sensitivity and specificity could arise from the timing of marker elevation, as certain biomarkers may only become detectable at later stages of the disease, leading to reduced sensitivity in the early phase. These findings emphasize the need for further research to understand the growth factors in preeclampsia.

Our study is the first to investigate the differences in various growth hormones in the maternal and cord blood. However, there are several limitations. Firstly, the study sample size was small and limited to Asian individuals, which may hinder generalization. Specifically, the collection of umbilical cord blood was challenging, leading to even fewer samples. Secondly, although we examined multiple factors simultaneously, the inability to confirm individual effects is a limitation. In addition the timing of sample collection is one of the limitations, as these markers need to be measured before the onset of preeclampsia. However, samples were taken once the disease had started or was already in progress. Therefore, future studies should focus on sampling before the onset of preeclampsia for further exploration. It is still unclear whether the elevated growth factors are a consequence of or a cause of preeclampsia. In addition our study was not designed to characterize to determine the expression of growth factors in placenta. Fourth, sFlt-1 in blood is one of the predictive markers for preeclampsia and is clinically assessed in women with the condition. However, the correlation of this marker could not be assessed alongside with growth factors in our study because the available data are limited especially in normotensive pregnancy. Lastly, due to variations in the timing of blood collection, we could not adjust for small differences resulting from this factor.

In conclusion, we confirmed an increase in EGF, HGF, LIF and SCF in preeclampsia. Particularly, EGF, NGF-beta and SCF were associated with the elevation of blood pressure, while LIF was associated with platelets and SCF with serum creatinine and ALT, respectively. Additionally, EGF showed a positive correlation with the urinary protein/creatinine ratio and inverse relationship to birthweight. These findings suggest a diverse hormonal association with factors related to preeclampsia. Additionally, the lack of fluctuation in growth factors within the umbilical vein suggests a protective mechanism by the placenta for the fetus, while the secretion of various growth factors by the placenta may represent a distinct and separate process. Further specific research is needed to determine whether the increase in EGF, HGF, LIF and SCF is a consequence of preeclampsia or if it indicates changes in factors associated with preeclampsia.

## Data Availability

The data can be available on resonable request by corresponding author.
